# Synthesis and Application of Kraft Lignin-Based Polyurethane Coatings for Functional Paper Packaging Materials

**DOI:** 10.3390/polym18070787

**Published:** 2026-03-25

**Authors:** Julia de Cristo Figueiredo, Fernando José Borges Gomes, Ericka Figueiredo Alves Redmond, Biljana Bujanovic, Roberto Carlos Costa Lelis, Mayara Felix Santana, Clayton Mickles

**Affiliations:** 1 Forest Products Department, Forestry Institute, Universidade Federal Rural do Rio de Janeiro, BR-465 km 7, Seropédica 23897-000, RJ, Brazil; lelis@ufrrj.br; 2 Department of Chemical Engineering, College of Environmental Science and Forestry, State University of New York, 1 Forestry Drive, Syracuse, NY 13210, USA; eredmond@esf.edu (E.F.A.R.); msanta13@syr.edu (M.F.S.); 3 Forest Products Laboratory, U.S. Department of Agriculture-Forest Service, 1 Gifford Pinchot Drive, Madison, WI 53726, USA; bujanovic@usda.gov (B.B.); clayton.mickles@usda.gov (C.M.)

**Keywords:** biopolymer, paper barrier, sustainability, renewable materials, bio-based coatings

## Abstract

The packaging sector presents a significant sustainability challenge, particularly due to the prevalence of plastic packaging. There is a growing interest in sustainable packaging alternatives. The main challenge is to develop packaging with comparable and competitive characteristics. In this context, this manuscript aims to evaluate the performance of lignin-based polyurethane applied as a coating on recycled linerboard. Industrial softwood kraft lignin was fully characterized in terms of purity, functional groups (FTIR and ^31^P NMR) and molecular weight (GPC). Aiming at coating applications, the lignin sample was solubilized in dimethyl sulfoxide (DMSO) and used as a polyol substitute in the reaction, replacing polyethylene glycol (PEG) at levels of 70%, 80%, and 90%. Subsequently, hexamethylene diisocyanate (HDI) was added to initiate polyurethane formation. After polymerization, the coating was applied in multiple layers onto the linerboard paper. Regarding water resistance, all applications demonstrated effectiveness. The lignin-based polyurethane coating improved the Cobb1800 with reductions in the range of 1147.4 to 1155.8 g/m^2^ compared to the uncoated paper. Water vapor permeability was reduced by more than 94%. In the evaluation of oil resistance, samples with three layers and 90% lignin replacement performed particularly well, achieving a high value in a kit test for oil and grease (kit test number 12). These results highlight a promising approach to paper-based packaging, with potential applications across a wide range of products.

## 1. Introduction

An ever-increasing number of people are becoming concerned about the use of non-renewable resources and waste management. These consumers hold a negative perception of traditional food packaging, such as glass, plastic, and aluminum [1]. One synthetic material commonly used in packaging systems is polyurethane (PU) [2], which is synthesized through the polyaddition reaction of polyols and isocyanates [3], typically derived from petroleum. In general, polyurethane, along with expanded polystyrene, polypropylene, and aluminum foil, serves as a thermal insulator in packaging systems [4].

Polyurethanes (PUs) are synthesized through the reaction between isocyanates and polyols, resulting in polymers with various functional groups, including hydroxyl (OH), amine (NH), urethane (NH–C=O), and ether (-O-) linkages [5]. The properties and structure of PUs can be influenced by several factors, allowing for tailoring materials with excellent mechanical strength, chemical stability, and water resistance. Key factors impacting PU characteristics include the types of isocyanates and polyols used, reaction temperature, choice of solvent, and the presence of additional additives [5,6].

In addition, the precursors used in the synthesis of polyols, such as ethylene and propylene oxides, are derived from petrochemical sources, which are limited and may be exhausted in the near future [7]. In this regard, natural and bio-based polymers offer a promising alternative to petroleum-derived reagents [6]. While bio-based polyols, especially those derived from vegetable oils, are commonly used in polyurethane production, concerns exist about potential competition with food resources [8]. In this context, kraft lignin presents a promising raw material for polyurethane synthesis [8,9,10,11].

Currently, approximately 75% of the world’s pulp is produced by the kraft process, totaling around 170 million metric tons of cellulosic pulp per year. As a by-product, the pulp and paper industries generate ~ 70 million tons of lignin per year, and it is generally used to generate energy for the pulp mill [12,13,14,15,16]. Due to technological advances, most mills have energy surplus. According to Argyropoulos et al. (2023) [16], the potential lignin that can be extracted in a modern plant, considering the surplus energy generated (40% outtake of lignin), is approximately 450,000 metric tons per year. As an aromatic polymer, kraft lignin holds significant potential for a wide range of applications. Therefore, in response to the growing need to enhance profitability and reduce the carbon footprint of the kraft pulp industry, its conversion into higher value-added products that fully exploit its potential has been extensively studied [17].

The kraft pulping process substantially modifies the lignin, resulting in heterogeneous structures that are still largely unknown [18]. Nevertheless, kraft lignin contains hydroxyl groups, including phenolic and aliphatic hydroxyl groups, which can react with isocyanate groups to form urethane bonds, although phenolic hydroxyl groups exhibit lower reactivity [19,20]. Therefore, the abundance, availability and type of hydroxyl groups in lignin are fundamental to polyurethane synthesis [21].

The aim of this research is to characterize technical lignin in order to assess its suitability for polyurethane synthesis and, subsequently, to propose a polyurethane coating with high substitution of polyethylene glycol by lignin for paperboard in order to promote water and oil barrier properties. By using kraft lignin as the primary source of polyol instead of petroleum-based alternatives, we are contributing to efforts to improve the sustainability of the packaging sector, as well as adding value to kraft lignin, which is currently a by-product of the industry.

## 2. Materials and Methods

### 2.1. Materials

Softwood recycled linerboard paper, sourced from a paper mill, was used as the base for coating. Softwood kraft lignin (KL) was supplied by a pulp mill. Both materials are commercial and were donated to the State University of New York–College of Environmental Science and Forestry (SUNY-ESF) (New York, NY, USA). The production of lignin-based polyurethane involved the use of hexamethylene diisocyanate (HDI), dimethyl sulfoxide (DMSO), dibutyltin dilaurate (DBTDL), and polyethylene glycol 400 (PEG), which were purchased from Fisher Scientific (Waltham, MA, USA). The research was conducted at the State University of New York—College of Environmental Science and Forestry (Department of Chemical Engineering) and the Federal Rural University of Rio de Janeiro (Lignocellulosic Biorefinery Laboratory).

### 2.2. Methods

#### 2.2.1. Lignin Characterization

The lignin content, including both the insoluble and soluble lignin fractions, was determined following NREL/TP-510-42618 [22]. The ash content of kraft lignin was analyzed in accordance with NREL/TP-510-42622 [23]. Based on these results, the lignin purity was inferred gravimetrically. Fourier Transform Infrared Spectroscopy, PerkinElmer Frontier FT-IR/NIR (PerkinElmer Inc., Shelton, CT, USA), was used to record the spectra of lignin and the synthesized polyurethane. The samples were dried at 40 °C in a vacuum oven before analysis. FTIR spectra were recorded using 32 scans in the range of 500–4000 cm^−1^ at room temperature. To assess the polydispersity of kraft lignin, its molecular weight was measured using an Agilent Gel Permeation Chromatography (GPC) SECurity 1200 system (Agilent Technologies, Santa Clara, CA, USA). Prior to GPC analysis, the kraft lignin was acetylated with acetic anhydride/pyridine [24], dissolved in tetrahydrofuran (THF) and filtered through 0.45 μm PTFE filters. For further lignin characterization, Phosphorus Nuclear Magnetic Resonance (^31^P NMR) was performed to quantify and classify hydroxyl groups in kraft lignin. The analysis was conducted using a Bruker AVANCE 500 MHz NMR spectrometer (Bruker BioSpin GmbH & Co. KG, Ettlingen, Germany) equipped with a 5 mm liquid N_2_ cooled broadband fluorine observation probe. To prepare the sample, a vacuum-dried lignin sample was precisely weighed (20 mg) and dissolved in 500 μL of pyridine/deuterated chloroform (CDCl_3_) (1.6:1, *v*:*v*) containing endo-*N*-hydroxy-norbornene-2,3-dicarboximide (NHND, 4.0 mg/mL) as an internal standard and chromium acetylacetonate [Cr(acac)_3_] (1.0 mg/mL) as a relaxation agent. After complete dissolution, 100 μL of 2-chloro-4,4,5,5-tetramethyl-1,3,2-dioxaphopholane (TMDP) was added as a phosphitylation reagent [25]. The ^31^P NMR experiment was conducted immediately after sample preparation. The acquisition parameters were a spectral width of 100 ppm, 0.99 s acquisition time, 25 s relaxation delay, and 64 scans.

#### 2.2.2. Lignin-Based Polyurethane Synthesis

Kraft lignin was solubilized in dimethyl sulfoxide (DMSO) for 30 min. All reactions were carried out in a beaker under constant magnetic stirring inside a fume hood under open conditions (without sealing). Hexamethylene diisocyanate (HDI) was subsequently added, providing the necessary NCO groups. This was followed by the addition of polyethylene glycol 400 (PEG) and dibutyltin dilaurate (DBTDL) as a catalyst. The reaction occurred at ~20 °C for approximately 60 min until the first layer application. All steps were conducted under continuous stirring. The polyols, PEG and kraft lignin provided the OH groups essential for polyurethane synthesis. The experiment tested three different substitution levels of PEG with kraft lignin, specifically 70%, 80%, and 90% by weight, with the samples referred to, respectively, as 70% KL, 80% KL and 90% KL, along with a control sample that contained no kraft lignin. The dosage of each reagent is available in Table 1.

To verify the formation of polyurethane, Attenuated Total Reflectance Fourier Transform Infrared Spectroscopy (ATR-FTIR) was performed for the PUs formed (control, 70% KL, 80% KL and 90% KL) after 24 h of synthesis, under the same conditions as described earlier.

Thermogravimetric analysis (TGA) was performed using a T550 TGA (Waters TM TA Instruments, New Castle, DE, USA) to evaluate the thermal stability and mass loss of the materials. PUs (~8 mg) were heated from room temperature to 600 °C at a rate of 10 °C/min under a nitrogen atmosphere.

#### 2.2.3. Coating Application

Approximately one hour after the initiation of polymerization, the formulations were immediately applied onto the recycled linerboard surface to avoid premature curing of the polyurethane system. This ensured adequate spreading and prevented excessive penetration through the paper structure. After polyurethane synthesis, the layers (1, 2, or 3) were applied consecutively without intermediate curing or drying steps. Then the coated papers were placed in an oven at 105 °C for 5 min and transferred to a room with controlled temperature (23 ± 1 °C) and humidity (50 ± 2 °C), as described by Figueiredo et al. (2026) [26]. The samples were conditioned under these conditions for 24 h prior to the physical tests. The coating was applied using an automatic coater, K Control Coater (RK PrintCoat Instruments Ltd., Cambridgeshire, UK), with variations in bar thickness and application speed to obtain samples with a consistent grammage (30 ± 3 g/m^2^ of coating). Samples with one, two, and three layers of coating were tested, including the control samples.

#### 2.2.4. Paper Characterization

The uncoated paper (original) as well as those coated with lignin–polyurethane (70% KL, 80% KL and 90% KL) and the control (polyurethane without lignin) were subjected to a series of analytical tests. Standardized procedures were followed, as specified in various TAPPI and ASTM standards. Grammage was measured according to TAPPI/ANSI T 410 om-23, while thickness was evaluated using TAPPI/ANSI T 411 om-21 [27]. Air resistance was tested by the Gurley method following TAPPI/ANSI T 460 om-21 [28]. Water absorptiveness was determined via the Cobb test, as per TAPPI/ANSI T 441 om-20 [29], and grease resistance was assessed according to TAPPI T 559 cm-22 [30]. The water vapor transmission rate was evaluated gravimetrically in accordance with ASTM E96/E96M-21 [31]. Surface wettability was measured using the contact angle method (TAPPI T 458 cm-14 [32]), and ring crush strength was tested using the rigid support method (ANSI/TAPPI T 822 om-22 [33]). Tensile properties were measured according to TAPPI/ANSI T 494 om-22 [34], and the bursting strength of the linerboard was tested following TAPPI T 807 om-16 [35].

#### 2.2.5. Scanning Electron Microscopy (SEM)

Scanning electron microscopy (SEM) was carried out to evaluate the surface of the coated and uncoated samples using Hitachi TM3000 equipment (Hitachi, Ltd., Tokyo, Japan) at 100× magnification, a data size of 640 × 520, 15,000 Volts and low vacuum. The paper samples that were subject to analysis were first acclimatized in a room with controlled temperature (23 ± 1 °C) and humidity (50 ± 2 °C).

#### 2.2.6. Statistics

Tukey’s test was applied to assess the variability in paper properties across all samples produced. This test allows for multiple comparisons between group averages, allowing the identification of which groups show significant differences after detecting overall variation among more than two groups by means of Analysis of Variance (ANOVA).

## 3. Results and Discussion

### 3.1. Lignin Characterization

A general understanding of lignin’s chemical characteristics is fundamental for optimizing polyurethane production, as it provides insight into its reactivity, compatibility and performance. The kraft lignin used in this study is of high purity, with lignin comprising 96.43% of the material as determined gravimetrically (acid-insoluble lignin, 92.88%) and through UV-Vis spectral analysis (acid-soluble lignin, 3.55%). The remaining components include 1.13% ash and unidentified constituents, most likely carbohydrates. These results align well with the chemical composition of LignoBoost kraft lignin reported by Hu et al. (2016) and Constant et al. (2016) [36,37].

The molecular weight distribution obtained through GPC provides insights into the size and molecular dispersion of lignin, which directly influence its reactivity. The sample exhibited a weight-average (Mw) molecular weight of 7956 g/mol, a number-average (Mn) molecular weight of 1073 g/mol, and a polydispersity index (Mw/Mn) of 7.4. These results are in agreement with the molecular weight distribution reported for LignoBoost, demonstrating a relatively high polydispersity of softwood kraft lignin as an inherent property [36]. The heterogeneous structure and high polydispersity of lignin pose significant challenges to its effective dispersion for PU production [38]. The heterogeneous dispersion of lignin in the polyurethane matrix results from its wide distribution of particle sizes. Large or irregularly sized particles tend to aggregate, reducing interaction with isocyanates during polymerization [39].

**Figure 1:**
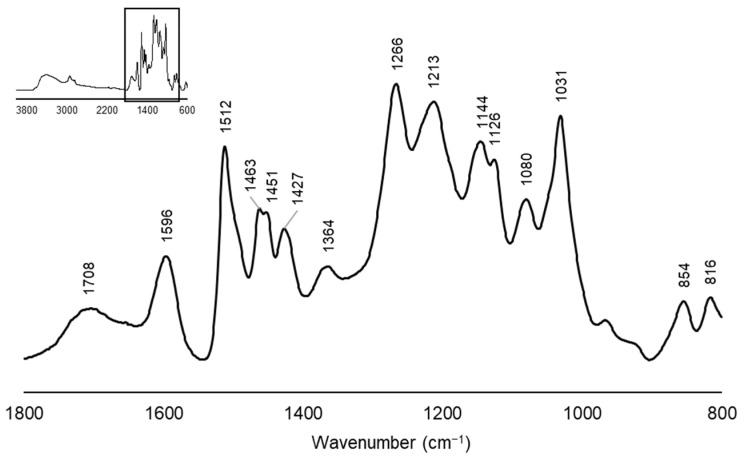
Normalized FTIR spectra of softwood kraft lignin.

The FTIR spectra of the softwood kraft lignin are shown in Figure 1. The band at 3500–3300 cm^−1^ is attributed to hydroxyl groups [40]. Furthermore, the bands at 2930 cm^−1^ and 2832 cm^−1^ in the spectra correspond to C-H stretching [41]. The band at 1708 cm^−1^ is associated with unconjugated carbonyl groups, C=O [42,43]. The band at 1596 cm^−1^ corresponds to aromatic skeletal vibrations combined with C=O stretching [44]. The bands at 1512 cm^−1^ and 1427 cm^−1^ are attributed to aromatic rings, while the band at 1463 cm^−1^ is related to C-H deformation. The bands at 1266 cm^−1^ and 1213 cm^−1^ correspond to guaiacyl C-O units and C-O guaiacyl vibration, respectively, and the band at 1031 cm^−1^ is linked to C-O-C stretching [42]. All the bands identified in the FTIR spectra are characteristics of softwood lignin, as shown in Figure 1.

**Figure 2:**
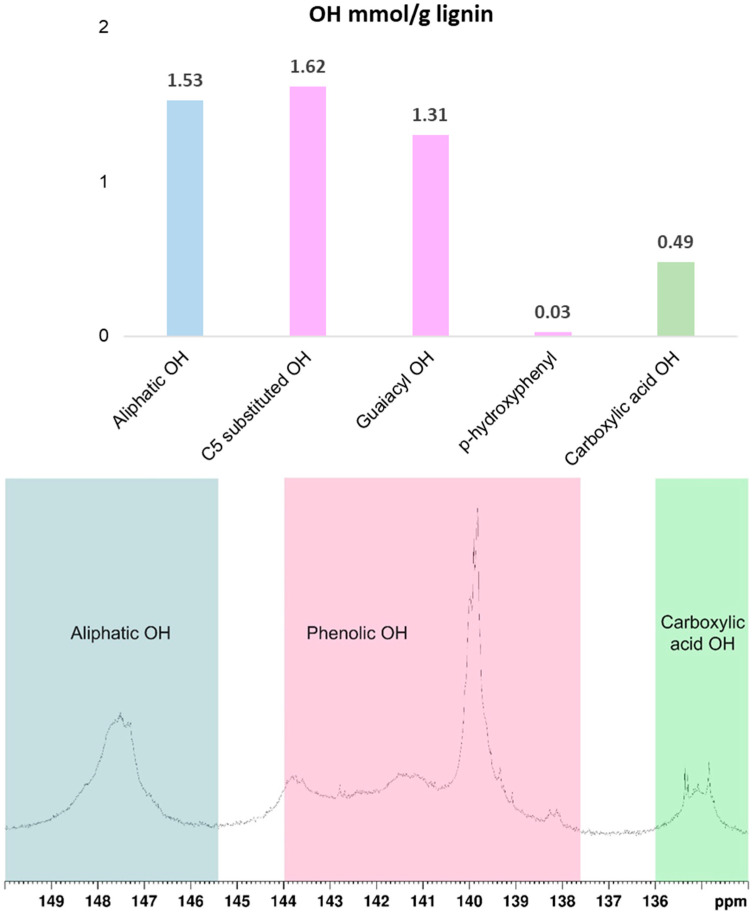
Functional lignin groups by quantitative ^31^P NMR measurements after phosphitylation.

Given the critical role of hydroxyl groups in PU production, the hydroxyl groups were quantified and classified using ^31^P NMR, which can be categorized into three main regions: 145.4–150 ppm (aliphatic OH), 137.6–144 ppm (phenolic OH), and 133.6–136 ppm (carboxylic acid OH). Figure 2 shows the quantification of these hydroxyl groups in softwood kraft lignin. Notably, the content of phenolic hydroxyl is higher than that of aliphatic hydroxyl groups. It has been observed that both hydroxyl (OH) groups react with HDI, but the reaction rate with aliphatic hydroxyl groups is faster. Aliphatic hydroxyl groups enhance the reactivity between lignin and HDI [45]. To improve the reactivity of phenolic hydroxyl groups, the addition of catalysts has been proposed, as they can either accelerate the reaction rate or reduce the dependence on aliphatic hydroxyl groups in the polyurethane formation process [46].

### 3.2. Polyurethane Characterization

**Figure 3:**
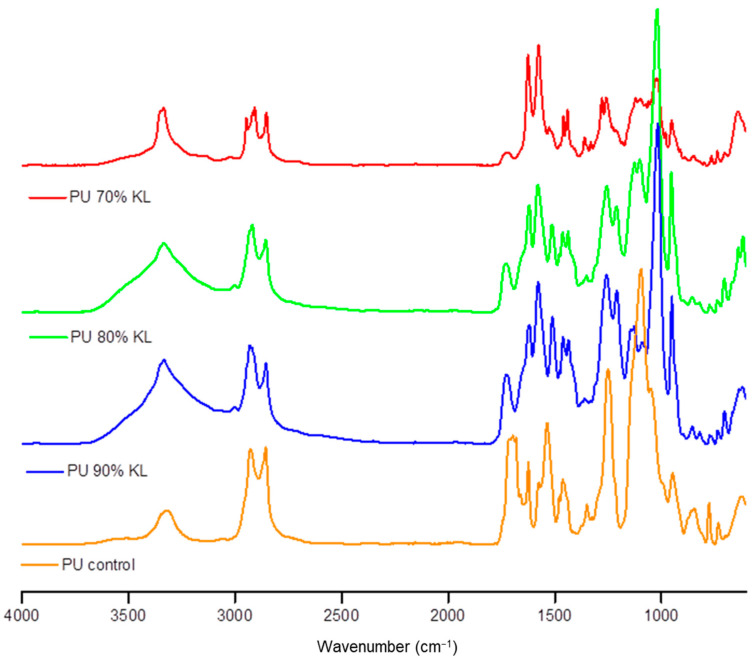
Normalized FTIR spectra of the synthesized polyurethanes: control, 70% KL, 80% KL and 90% KL.

FTIR was performed to confirm the chemical structure of the synthesized polyurethanes, as shown in Figure 3.

In FTIR spectroscopy, broad signals in the region of 3445–3255 cm^−1^ were attributed to N–H stretching, consistent with urethane bond formation [47]. These observations align with Griffini et al. (2015) [48], who reported a similar N–H signal (~3370 cm^−1^) emerging as lignin reacts with TDI. The C–H stretching vibrations observed at 2920 and 2850 cm^−1^ correspond to aliphatic chains introduced during polyurethane synthesis [8]. The sharp band at 1720 cm^−1^ is indicative of C=O stretching in urethane groups, distinguishing it from the weaker carbonyl signal (~1708 cm^−1^) in lignin [48]. The band around 1090 cm^−1^ confirms the presence of C-O stretching. Additional confirmation of urethane linkage formation was provided by N–H bending bands at 1655 and 1578 cm^−1^ [47], and a progressive increase in the C–N stretching band (~1207–1250 cm^−1^), which intensified with higher lignin content. Simultaneously, a decrease in the phenolic O–H band (~1365 cm^−1^) suggests covalent bonding between lignin and isocyanate [48]. The absence of the isocyanate NCO band (~2270 cm^−1^) indicates complete consumption of the diisocyanate, reinforcing the efficiency of crosslinking. Interestingly, a distinct band at 1016 cm^−1^, absent in the control but present in lignin-containing PUs, may reflect residual DMSO or associated structural changes during reaction. The characteristic DMSO band (~1050 cm^−1^) was also absent. Collectively, these spectral features confirm the successful synthesis of lignin-based polyurethane networks.

Hydrogen-bonded carbonyl and free amide groups are typically associated with the rigid segments of polyurethane networks. The relative intensity of these bands can provide insight into urethane bond formation [49]. In this study, the isocyanate content was kept constant, while the variation in PEG and kraft lignin proportions directly influenced the hydroxyl content and, consequently, the NCO/OH ratio. Given the variable reactivity of the hydroxyl groups in lignin, increasing the substitution of PEG for lignin in the formulation may result in higher NCO/OH ratios. This is mainly due to the lower functionality and reactivity of lignin, especially in relation to its phenolic OH groups. Although these groups can react with aliphatic diisocyanates such as HDI, their conversion is generally incomplete under mild reaction conditions [50]. Additionally, isocyanate may react with hydroxyl groups on cellulose substrates, potentially forming covalent bonds that further integrate the polymer with the paper surface and reinforce the overall network architecture [6,51].

**Figure 4:**
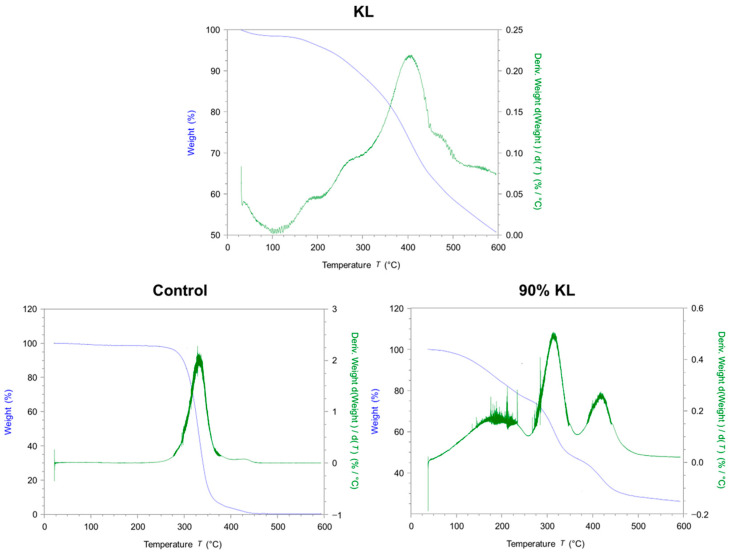
Thermogravimetric analysis (TGA) and derivative thermogravimetry (DTG) curves of the studied samples under a nitrogen atmosphere.

The thermal behavior of the synthesized polyurethanes was further evaluated by thermogravimetric analysis (TGA), as shown in Figure 4.

Thermogravimetric analysis (TGA) and its derivative (DTG) revealed the thermal degradation profiles of the samples (KL and synthesized Pus). KL exhibited a weight loss of approximately 50% by 600 °C, in agreement with the literature [52]. TGA and DTG profiles of the samples reveal distinct thermal behaviors associated with their chemical structures and aromatic content.

The kraft lignin used in this study was the same as in the previous work [26]. Its thermal degradation behavior is consistent with previous reports. The first mass loss observed in the DTG corresponds to water evaporation or moisture reduction. Between 100 and 300 °C there was a reduction in weight due to the degradation of carbohydrates. The main degradation peak observed in the DTG occurred in the temperature range of approximately 300 to 525 °C, with the most significant degradation at ~400 °C [52].

In contrast, the control sample exhibits a well-defined degradation event around 300 °C, attributed to the breakdown of urethane linkages and the degradation of flexible PEG chains, and leads to minimal char formation. When PEG is partially replaced by KL, the degradation profile becomes more complex and begins at a lower temperature. A peak around 200 °C is attributed to the evaporation of residual DMSO remaining in the system. The subsequent peak near 325 °C corresponds to the decomposition of urethane bonds, while the event around 425 °C is associated with the breakdown of aromatic rings and the onset of primary oxidative decomposition of unreacted lignin [52].

These results suggest the successful synthesis of PU. KL incorporation into the polyurethane matrix reduces initial thermal stability but increases the overall complexity of the degradation process and enhances char yield. These modifications may be advantageous for specific applications requiring thermal resistance and residue formation.

### 3.3. Paper Characterization

As illustrated in Table 2, the averages of the grammage and thickness values were determined for the original paper (uncoated), the control (polyurethane-coated paper), and the lignin-based polyurethane-coated papers at 70%, 80%, and 90% substitution levels. Since grammage and thickness directly influence the physical properties of the paper, maintaining consistency across samples was essential to ensure reliable comparisons.

**Figure 5:**
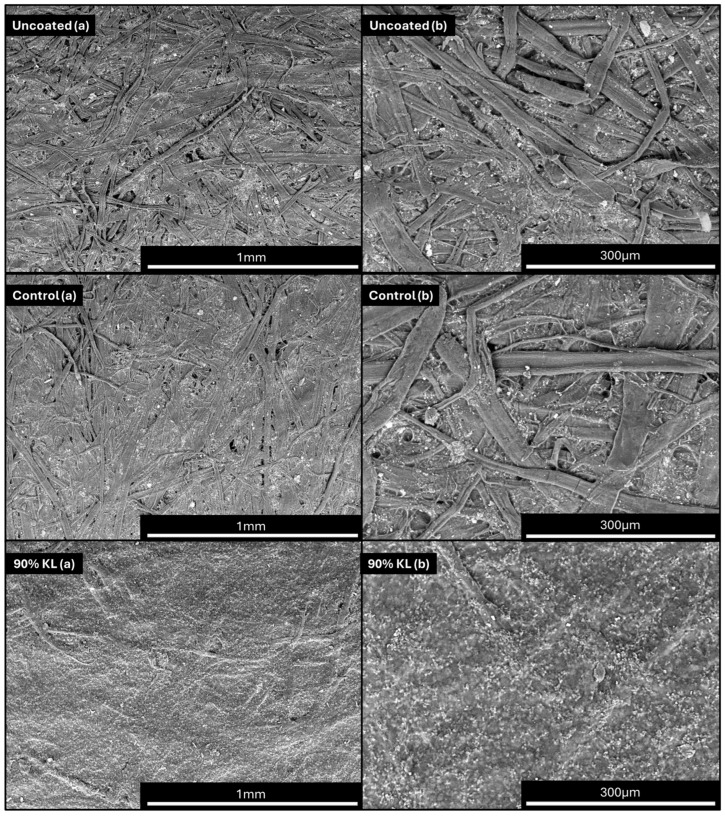
Scanning electron microscopy (SEM) micrographs of paper samples coated with different formulations at two magnifications. For each sample, images labeled (**a**) correspond to 100× magnification, while images labeled (**b**) correspond to 300× magnification.

The uncoated paper (original) exhibited the lowest grammage and thickness, as expected. The control samples with two and three layers exhibited slightly high grammage due to the difficulty of applying multiple layers of polyurethane in a manner that uniformly covers the paper. However, there was no statistical variation in the thickness among the control samples at a 5% significance level. In contrast, all lignin-based polyurethane-coated papers displayed similar grammage and thickness. In addition to these findings, it was evident that the coating not only formed a layer on the paper surface but also penetrated the spaces between the fibers, serving as an adhesive to bind them together [53]. This can be observed in SEM images (Figure 5).

**Figure 6:**
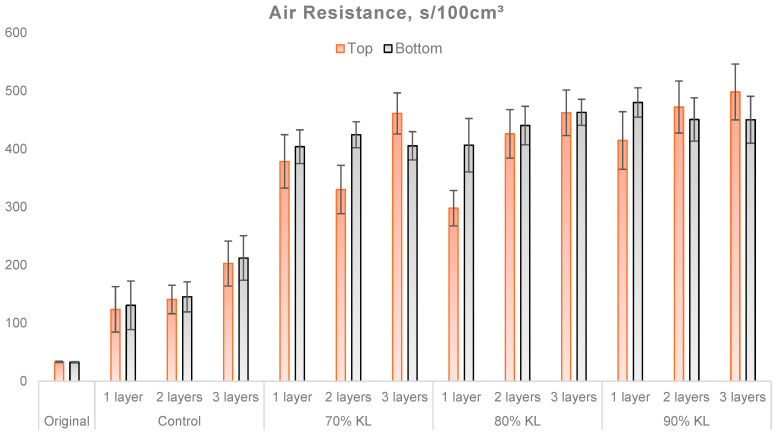
Results for air resistance of uncoated and coated papers.

The grammage and thickness of a paper provide fundamental information about density and composition. However, air permeability reveals important aspects of porosity and internal bonding structure. For applications that require robust barriers against gases, such as food and industrial packaging, papers with low air permeability are more advantageous. Air permeability plays a critical role in determining a material’s barrier properties [54]. Figure 6 shows the average air resistance of the different samples, considering variations in the number of layers and the type of coating.

The original sample (uncoated) showed the lowest resistance to the passage of air. Among the coated samples, the 90% KL formulation demonstrated the best performance, with values of 498 s/100 cm^3^ on the top side and 450 s/100 cm^3^ on the bottom side for three layers of coating. In contrast, the control samples showed the lowest results, with values of 124 s/100 cm^3^ on the top side and 131 s/100 cm^3^ on the bottom side with a single layer of coating applied.

Despite the similarity in grammage and thickness among the evaluated samples, those with a greater number of layers exhibited more homogeneous coverage. This improvement can be attributed to the additional layers correcting imperfections in the preceding layers. Consequently, this process resulted in a more uniform paper surface with a better cover finish.

Tukey’s statistical analysis at a 5% significance level for air resistance indicated that the original sample differed significantly from all others. This property increased by 269% to 549% in the control samples across both test directions. In contrast, the 70% KL, 80% KL, and 90% KL samples exhibited a 790% to 1387% increase compared to the original sample in both directions. As expected, the application of the coating and the increase in layers led to a reduction in the paper’s air permeability.

**Figure 7:**
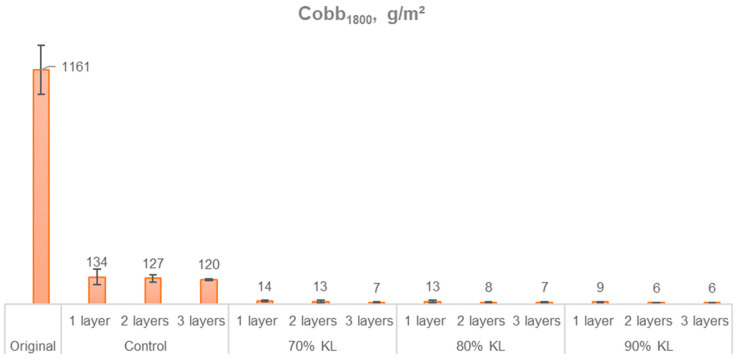
Water absorptiveness of paper.

The combined analysis of air permeance and water absorption, as measured by the Cobb test, allows for a more comprehensive understanding of the paper’s functional properties. The homogeneity and composition of the coating are determining factors in optimizing the paper’s performance in these tests. Figure 7 presents the average of the Cobb_1800_ test for each sample.

The original and control samples showed inferior results in the Cobb_1800_ test, indicating higher water absorption. The graph shows a downward trend in water absorption as more layers of coating are applied. This is due to the formation of a more uniform coating, which creates a more effective barrier against liquid penetration. The lignin coating creates a more resistant barrier, allowing less water to penetrate after 1800 s of testing. This outcome is potentially associated with the formation of urethane bonds, promoted by the reaction of HDI with lignin, thereby effectively increasing the hydrophobicity of the PU films [55].

The coating application reduced water absorptiveness by 88.4% to 89.6% in the control samples and by 98.8% to 99.5% in the 70% KL, 80% KL, and 90% KL samples. Among these three coated samples, no statistically significant differences were observed at a 5% significance level in the Tukey test. As expected, in addition to the inherent hydrophobicity of the polyurethane, its application to the paper also forms a physical barrier, effectively limiting the penetration of water through the layers until it reaches the paper, which is hygroscopic.

**Figure 8:**
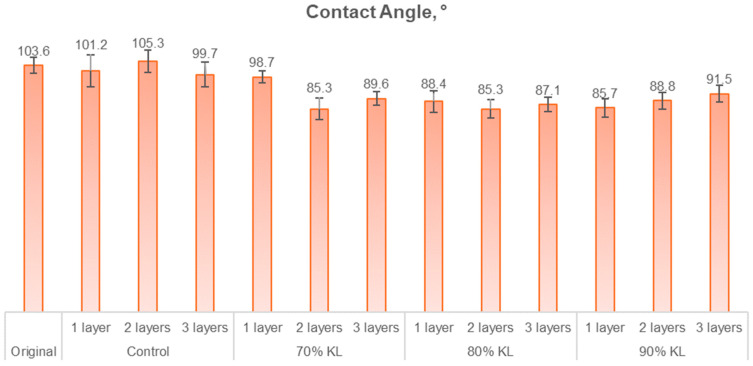
Contact angle of uncoated and coated papers.

An additional analysis employed to assess the water resistance of the paper is that of the contact angle. This analysis did not demonstrate the same trend as the Cobb, which is reported in Figure 8. This discrepancy can be attributed to the surface tension of the paper, with the contact angle being measured in the first few seconds after the drop is exposed to the surface of the paper and before it stabilizes, typically between 0.25 and 3 s.

Statistical analysis of the contact angle results revealed that the control samples with one, two, and three layers exhibited no significant differences between each other, or between these control samples and the original sample or the 70% lignin sample with one layer. Additionally, the control sample with three layers showed no significant statistical difference when compared to the 90% lignin sample with three layers. Among the lignin-containing samples, the only one that showed a significant difference from the others, except for the 90% lignin sample with three layers, was the 70% lignin sample with one layer. All other samples were found to be statistically equivalent. The reduction in the property for lignin-based polyurethane samples varied between 4.7% and 17.7%. This unexpected result could be caused by the residual DMSO present in the sample, as it exhibits high polarization, is an aprotic solvent, and contains apolar groups, contributing to its amphipathic nature [56,57].

**Figure 9:**
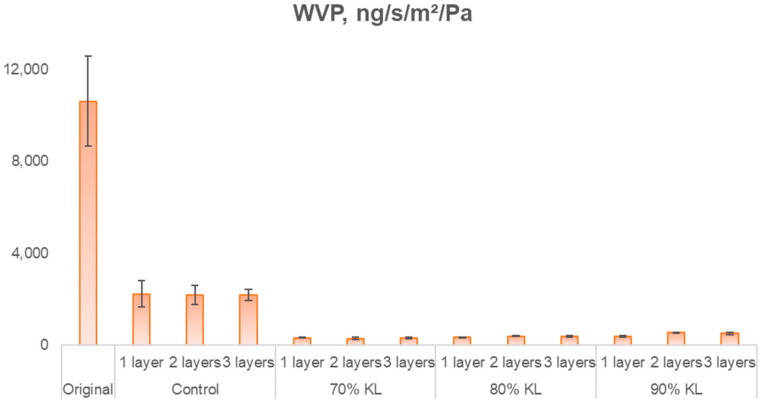
Water vapor permeance of uncoated and coated papers.

Although contact angle analysis characterizes the surface wettability and interfacial energy of the coated linerboard, the Cobb value constitutes a more functionally relevant parameter in the context of packaging applications, as it quantitatively reflects the overall water absorption behavior of the fibrous network under dynamic exposure conditions. In this sense, the water vapor transmission rate (WVTR) further complements the barrier evaluation by measuring the permeance of water vapor through the paper structure. The results are reported as water vapor permeance, as shown in Figure 9. Water vapor permeability can be correlated with air resistance and Cobb values, since all three parameters are influenced by coating continuity, porosity reduction, and restriction of moisture transport pathways. Collectively, these results consistently demonstrated that the lignin-containing coating provided superior barrier efficiency compared to the lignin-free formulation.

Tukey’s statistical analysis at a 5% significance level of water vapor permeance indicated that the lignin-containing samples did not differ significantly from one another. Similarly, the control samples were statistically similar and showed no difference from the 90% KL with two and three layers. In contrast, the original sample exhibited a statistically significant difference from all other samples. The reductions in WVTR for the lignin-based coating and the original sample were over 95%, exhibiting a great improvement in this property.

**Figure 10:**
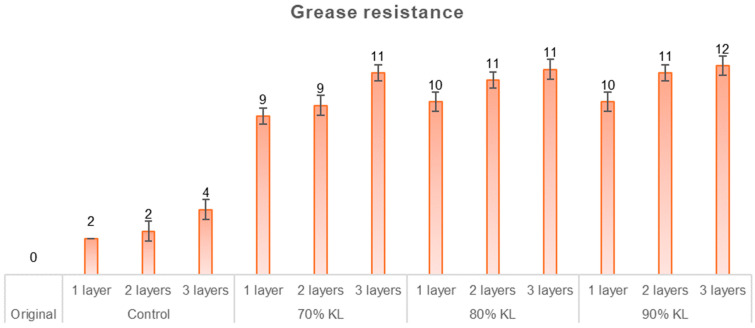
Grease resistance test of uncoated and coated papers.

The results from the air permeance and the water resistance tests provide valuable insights into how paper reacts to liquid penetration in general. However, oil and grease resistance tests are essential to assess the coating’s ability to block non-aqueous substances. These substances can exhibit varying penetration behaviors due to their distinct chemical and physical properties, which in turn can affect the barrier properties of the coating, depending on factors such as the type of film used and its thickness [58]. The kit oil test results, shown in Figure 10, illustrate an improvement in oil resistance properties as the number of layers increases, with all samples demonstrating a consistent trend of improvement.

An increase in lignin content in the formulation, combined with a greater number of applied layers, resulted in more pronounced improvements in oil resistance. Statistical analysis revealed that the samples 90% KL with two and three layers, 80% KL with two and three layers, and 70% KL with three layers exhibited comparable performance. In general, all samples with lignin-based polyurethane demonstrated superior resistance to oil and grease penetration compared to the original and control samples, making them more suitable for use in packaging that requires protection against oily substances.

### 3.4. Paper Strength

**Figure 11:**
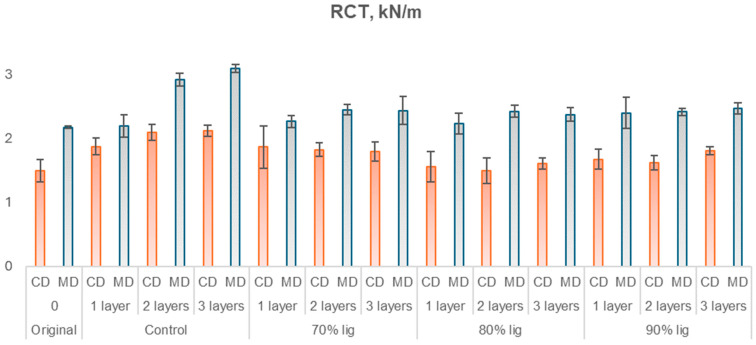
Ring crush of uncoated and coated papers in CD and MD.

**Figure 12:**
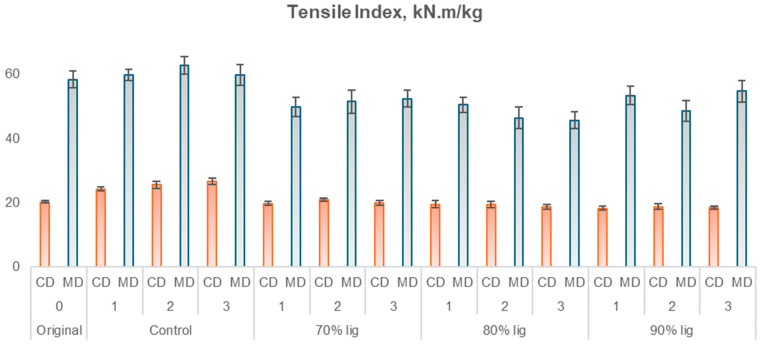
Tensile index of uncoated and coated papers in CD and MD.

**Figure 13:**
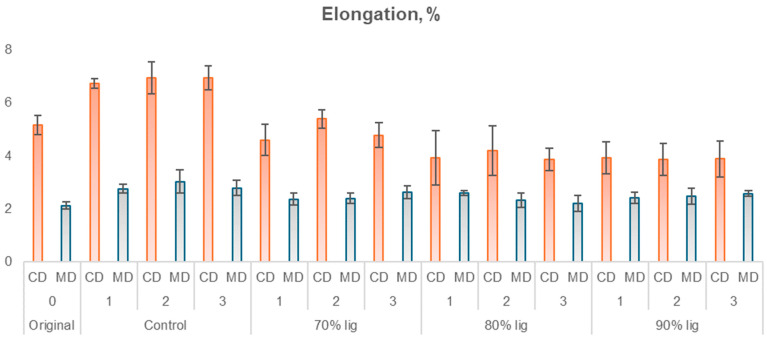
Elongation of coated and uncoated papers in CD and MD.

**Figure 14:**
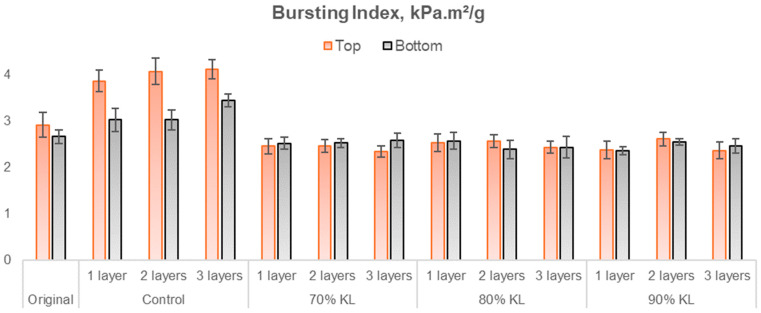
Burst index of coated and uncoated papers.

As the studied material is linerboard intended for packaging applications, it is essential to conduct mechanical tests typically used in the industry. Considering the industrial-grade nature of the linerboard, the tests were performed in both the machine direction (MD) and cross direction (CD) as well as at the top and bottom of the paper, to evaluate the bursting index. The results of these tests are presented in Figure 11, Figure 12, Figure 13 and Figure 14, which correspond to the ring crush test, tensile index, elongation, and burst index, respectively.

The ring crush test (RCT) results (Figure 10) demonstrated statistical similarity among most samples at a 5% significance level when analyzed in the machine direction. Among all the samples tested, only the control samples with two and three layers showed significant differences from the others, while the remaining samples were statistically similar. In relation to the cross-machine direction, the original sample showed statistical similarity at a 5% significance level with the samples 80% KL with one, two, and three layers, as well as with the samples 90% KL with one and two layers. All other samples differed significantly from each other. All coated samples present higher compression resistance compared to the original. Control samples had a gain in compression resistance from 1.2% to 42.2%, while lignin-based polyurethane-coated papers had 0.1% to 25.1%. Due to the high polydispersity and structural heterogeneity of kraft lignin, polyurethane formation may not have occurred uniformly throughout the system, potentially leading to localized differences in network formation, which may influence compressive resistance. The slight increase can be attributed to the intrinsic rigidity of lignin molecules [59,60].

Among the tensile indexes (Figure 11), in the cross-machine direction, the tensile test revealed that the original sample differed significantly, at a 5% significance level, from all control samples and the sample containing 90% lignin with one layer. In the machine direction, the original sample differed from the samples with 80% lignin (one, two, and three layers), 70% lignin with one layer, and 90% lignin with two layers. While control samples exhibited, in both directions, an increase in tensile resistance (0.71% to 31.6%), lignin-based PU samples showed, in both directions, a decrease of 1.5% to 21.7%, except 70% KL with two layers in the CD, which had an increase of 3.2%.

Again, the increase in lignin content could contribute to this result. In addition, the difference in behavior between TI and RCT is because compression forces tend to be distributed more evenly throughout the sample, while in the tensile test, the concentration of tensions at specific points, such as defects or discontinuities in the paper structure, can lead to rupture. The absence of significant improvement in the tensile index suggests that the reinforcing effect of the lignin-based polyurethane coating was balanced by structural factors within the paper network. The broad molecular weight distribution and heterogeneous reactivity of kraft lignin may promote uneven network formation, while partial polymer penetration into the fiber matrix can alter inter-fiber bonding [26].

Analyzing the results in Figure 12 for elongation in the cross-machine direction, the original sample exhibited statistical significance at the 5% significance level, differing only from the control samples with one, two, and three layers. This finding suggests that the application of the coating did not affect the bonding properties between the fibers. A similar pattern was observed in the machine direction. All coated samples had higher elongation in the machine direction. The variation in the coated samples in relation to the original sample ranged from 3.4% to 43.0%, with the control samples exhibiting the largest increases in this property. In contrast, the CD exhibited an increase of 30% to 34% for control samples and a decrease for lignin-based PU samples (7.2% to 25.0%), except for 70% KL with two layers, which gained 4.5% in elongation. This property is directly affected by the rigidity of the material.

When the modulus of elasticity of the samples was evaluated, no statistical difference was observed in the cross-machine direction, except for the original sample and the one containing 80% lignin with one layer. However, in the machine direction, all lignin-containing samples differed statistically from the original. All samples in the CD exhibited an increase ranging from 6.7% to 44.7%, while those in the MD showed a decrease of 6.5% to 23.7%. The increase in this property indicates greater material rigidity compared to the original. The highest increases were observed in lignin-based polyurethane samples, which aligns with the high rigidity of lignin molecules in polyurethane formulations.

In the burst test results (Figure 13), the original sample was statistically similar to the samples with 70% lignin and two layers, 80% lignin with one and two layers, and 90% lignin with two layers on the upper surface of the paper (top). However, on the lower surface (bottom), the original sample differed statistically only from the control sample with three layers. The percentages of increase for control samples varied from 13.5% to 40.8% and decreased for lignin-based PU from 3.2% to 19.7% when considering both test directions.

In summary, the lignin-based coatings exhibited a slight reduction in mechanical performance, except for RCT, while the control sample, which did not contain lignin, showed a decrease in MOE. These variations can be explained by the high lignin PDI, which may have introduced significant heterogeneity in the polymer matrix. Given the inherently high polydispersity of kraft lignin, the reactivity of its hydroxyl groups can vary considerably, leading to less uniform crosslinking. As a result, the final coating may exhibit localized differences in network density and mechanical integrity, ultimately affecting the homogeneity of resistance properties on the paper surface.

The findings of the present study are consistent with those reported in our previous work [26], in which kraft lignin was also successfully incorporated into polyurethane coatings for recycled linerboard. In both studies, lignin acted as a functional bio-based polyol, contributing to improved coating continuity and significant enhancement of barrier performance compared to uncoated substrates.

## 4. Conclusions

This study evaluated the barrier properties of paper coated with lignin-based PU with lignin at varying substitution levels for PEG. The findings suggest that samples with the highest lignin content as polyol for polyurethane synthesis (90% KL) and three layers demonstrated the best performance in the oil and grease barrier, air resistance and water tests, but not the contact angle test. Grammage and thickness directly influenced the paper’s air permeability. Adding layers of coating resulted in more uniform coverage, enhancing its effectiveness as a barrier against liquids and gases. Cobb tests revealed that samples with lignin coatings exhibited lower water absorption, which is particularly relevant for packaging applications requiring high resistance to liquid penetration. In the mechanical tests, the samples with lignin-based coatings did not improve in performance.

In conclusion, substituting PEG with lignin in the synthesis of a PU coating effectively improved the evaluated paper’s barrier properties, but due to concerns regarding DMSO toxicity, future studies should include migration testing to quantify potential solvent residues and evaluate the safety of the final material for human-related applications.

## Figures and Tables

**Table 1:** Amounts of chemical reagents employed in the synthesis of lignin-based polyurethane.

Chemicals	Control	70% KL	80% KL	90% KL
Lignin (g)	-	7.0	8.0	9.0
DMSO (mL)	-	16.8	19.2	21.6
HDI (g)	6.0	6.0	6.0	6.0
PEG (g)	10.0	3.0	2.0	1.0
DBTDL (g)	0.015	0.015	0.015	0.015

**Table 2:** Grammage and thickness results.

Sample		Grammage (g/m^2^)	Thickness (µm)
Original		167.7 ± 1.1	246 ± 4
Control	1 layer	197.1 ± 2.7	249 ± 4
	2 layers	199.7 ± 1.5	248 ± 3
	3 layers	204.8 ± 2.2	250 ± 2
70% KL	1 layer	198.3 ± 1.7	268 ± 3
	2 layers	197.3 ± 1.7	265 ± 5
	3 layers	198.6 ± 1.9	267 ± 5
80% KL	1 layer	196.7 ± 3.9	267 ± 4
	2 layers	195.7 ± 2.5	266 ± 2
	3 layers	195.9 ± 3.0	266 ± 3
90% KL	1 layer	197.6 ± 4.0	267 ± 4
	2 layers	196.7 ± 2.9	268 ± 4
	3 layers	196.7 ± 2.2	266 ± 3

## Data Availability

The raw data supporting the conclusions of this article will be made available by the authors on request.

## Abbreviations

The following abbreviations are used in this manuscript:

KL	Kraft Lignin
PU	Polyurethane
DMSO	Dimethyl Sulfoxide
PEG	Polyethylene Glycol 400
DBTDL	Dibutyltin Dilaurate
HDI	Hexamethylene Diisocyanate
FTIR	Fourier Transform Infrared Spectroscopy
GPC	Gel Permeation Chromatography
^31^P NMR	Phosphorus Nuclear Magnetic Resonance
SEM	Scanning Electron Microscopy
